# Optimal amino acid system for early embryo development in sows based on response surface methodology and high-throughput screening cell models

**DOI:** 10.1186/s40104-025-01194-w

**Published:** 2025-04-25

**Authors:** Xinyu Wang, Jun Huang, Yanlong Li, Zhekun Zhu, Bangxin Xue, Yueyang Meng, Jiale Bao, Ran Ning, Siyu Li, Fang Chen, Shihai Zhang, Xiangzhou Zeng, Shuang Cai, Chuanjiang Cai, Xiangfang Zeng

**Affiliations:** 1 Frontier Technology Research Institute of China Agricultural University in Shenzhen, Shenzhen, 518116 China; 2 State Key Laboratory of Animal Nutrition and Feeding, Ministry of Agriculture and Rural Affairs Feed Industry Centre, China Agricultural University, No. 2 Yuanmingyuan West Road, Beijing, 100193 China; 3 Beijing Key Laboratory of Biofeed Additives, China Agricultural University, Beijing, 100193 PR China; 4 Guangdong Province Key Laboratory of Animal Nutrition Control, College of Animal Science, South China Agricultural University, Guangzhou, 510642 China; 5 College of Animal Science and Technology Northwest, A&F University, Yangling, 712100 China

**Keywords:** Amino acid, Embryonic development, High throughput screening, Nutrient, Response Surface Methodology

## Abstract

**Background:**

Early embryo development plays a pivotal role in determining pregnancy outcomes, postnatal development, and lifelong health. Therefore, the strategic selection of functional nutrients to enhance embryo development is of paramount importance. In this study, we established a stable porcine trophectoderm cell line expressing dual fluorescent reporter genes driven by the *CDX2* and *TEAD4* gene promoter segments using lentiviral transfection.

**Results:**

Three amino acid metabolites—kynurenic acid, taurine, and tryptamine—met the minimum z-score criteria of 2.0 for both luciferase and Renilla luciferase activities and were initially identified as potential metabolites for embryo development, with their beneficial effects validated by qPCR. Given that the identified metabolites are closely related to methionine, arginine, and tryptophan, we selected these three amino acids, using lysine as a standard, and employed response surface methodology combined with our high-throughput screening cell model to efficiently screen and optimize amino acid combination conducive to early embryo development. The optimized candidate amino acid system included lysine (1.87 mmol/L), methionine (0.82 mmol/L), tryptophan (0.23 mmol/L), and arginine (3 mmol/L), with the ratio of 1:0.43:0.12:1.60. In vitro experiments confirmed that this amino acid system enhances the expression of key genes involved in early embryonic development and improves in vitro embryo adhesion. Transcriptomic analysis of blastocysts suggested that candidate amino acid system enhances early embryo development by regulating early embryonic cell cycle and differentiation, as well as improving nutrient absorption. Furthermore, based on response surface methodology, 400 sows were used to verify this amino acid system, substituting arginine with the more cost-effective *N*-carbamoyl glutamate (NCG), a precursor of arginine. The optimal dietary amino acid requirement was predicted to be 0.71% lysine, 0.32% methionine, 0.22% tryptophan, and 0.10% NCG for sows during early gestation. The optimized amino acid system ratio of the feed, derived from the peripheral release of essential amino acids, was found to be 1:0.45:0.13, which is largely consistent with the results obtained from the cell model optimization. Subsequently, we furtherly verified that this optimal dietary amino acid system significantly increased total litter size, live litter size and litter weight in sows.

**Conclusions:**

In summary, we successfully established a dual-fluorescent high-throughput screening cell model for the efficient identification of potential nutrients that would promote embryo development and implantation. This innovative approach overcomes the limitations of traditional amino acid nutrition studies in sows, providing a more effective model for enhancing reproductive outcomes.

**Supplementary Information:**

The online version contains supplementary material available at 10.1186/s40104-025-01194-w.

## Introduction

Embryo development significantly influences pregnancy outcomes, postnatal development, and lifelong health [1, 2]. Enhancing early embryo development and survival is thus a pivotal strategy for improving the overall reproductive performance of sows. There is growing evidence that maternal nutrition profoundly affects the quality and early development of mammalian embryos, with amino acids playing a crucial role in early embryonic development [3–5].

Numerous amino acids and their metabolites have been reported to influence early embryonic development [6, 7]. Methionine, an essential amino acid, is indispensable for DNA synthesis and cell proliferation, making it crucial for embryo development [8, 9]. Arginine, involved in nitric oxide synthesis and polyamine metabolism, modulates maternal nutrient metabolism and immune responses [10, 11]. Several studies suggest that arginine supplementation can improve reproductive performance in sows, chickens, and ruminants [12–14]. Additionally, serotonin, a metabolite of tryptophan, functions as both a neurotransmitter and a growth factor, with research indicating its role in regulating early embryo development in pigs through epigenetic modifications [15]. Overall, current research on the nutritional regulation of early embryo development in sows primarily focuses on the requirements of individual amino acids or their metabolites, with a lack of studies on their optimal composition and ratios [16–19]. Several challenges hinder systematic studies in sows, including the large size of the animals, high research costs, small experimental populations, and difficulties in data collection. Moreover, existing research approaches are time-consuming and inefficient in identifying and uncovering new potential amino acids or metabolites that promote early embryonic development in mammals [20]. Therefore, establishing new efficient approaches to optimize the amino acid profile for early embryo development in sows holds significant promise for enhancing reproductive performance in sows.

The aim of this study was to develop a dual-fluorescence high-throughput screening cell model that can effectively identify amino acids and their metabolites that regulate early embryonic development in sows. Additionally, we sought to determine an ideal amino acid system for early pregnancy using both in vivo and in vitro experiments combined with response surface methodology. The innovative approach proposed in this study overcomes the limitations of traditional research on amino acid nutrition requirement in sows and provides a more effective model for improving reproductive outcomes.

## Materials and methods

### Cell culture

The pTr cells (porcine trophectoderm cell line) were previously described and were kindly provided by Wu's research group, China Agricultural University [21]. The pEECs (porcine endometrial stromal cell line, Catalogue number: BFN60808569) were obtained from Qingqi Biotechnology Development Co., Ltd. (Shanghai, China). Both pTr and pEECs were cultured in DMEM supplemented with 10% fetal bovine serum, 100 U/mL penicillin, and 100 μg/mL streptomycin. The cells were maintained at 37 °C in a humidified atmosphere with 5% CO_2_, with subculture performed every 1–2 d.

### Construction of the luciferase reporter plasmids

Swine genomic DNA was extracted from pTr cells using the Universal Genomic DNA Extraction Kit (Takara, Kyoto, Japan). *CDX2* (Caudal Type Homeobox 2) and *TEAD4* (TEA Domain Transcription Factor 4) gene promoters were amplified using Phanta Max Master Mix (Vazyme, Nanjing, China) with various forward primers and a common reverse primer (Table 1). PCR products were cloned into the pGL4.17[luc2/Neo] luciferase vector (Promega, Madison, WI, USA) via InFusion Cloning (Vazyme, Nanjing, China), and confirmed by Sanger sequencing. Plasmids were propagated in *E. coli* DH5α cells (TIANGEN, Beijing, China) and purified using the TIANprep Mini Plasmid Kit for transfection.

**Table 1:** Primers used for vector

Name	Sequence (5′→3′)	Size, bp
	**Promoter amplification**	
*Promoter-CDX2-210*	AAGAAAGAGAGGGAGGGAGG	210
*Promoter-CDX2-315*	CTCGTTAATCACGTAAGGCCG	315
*Promoter-CDX2-521*	AGTGCACCAAGGTTGGAAGG	521
*Promoter-CDX2-1027*	TTCTACCACCCGCTTCTTGG	1,027
*Promoter-CDX2-1313*	CCATCCCCTGCAGATGAAAAAG	1,313
*Promoter-CDX2-1992*	TCCAGTCTCCCCCAGTCAAC	1,992
*Promoter-CDX2-R*	AGGTCCCGACGGCAAACCTCA	-
*Promoter-TEAD4-208*	GATGACCTGCCCTTGACCAG	208
*Promoter-TEAD4-333*	ACATCCATGGTACTTGGCCC	333
*Promoter-TEAD4-589*	TGTCCATGGACACTTGGGTT	589
*Promoter-TEAD4-989*	AGCTCCGATTTGACCTAGGC	989
*Promoter-TEAD4-1542*	CATATGGAGGTTCCCAGGCT	1,542
*Promoter-TEAD4-1998*	GCTATAGCTGCCGGCCTAC	1,998
*Promoter-TEAD4-R*	GTGGGCAGAAAGCACAGCAT	-
	**Vector linearization**	
*pGL-CDX2-210-R*	CTCCCTCTCTTTCTTGGTACCGGCCAGTTAGGC	
*pGL-CDX2-315-R*	TACGTGATTAACGAGGGTACCGGCCAGTTAGGC	
*pGL-CDX2-521-R*	CAACCTTGGTGCACTGGTACCGGCCAGTTAGGC	
*pGL-CDX2-1027-R*	AAGCGGGTGGTAGAAGGTACCGGCCAGTTAGGC	
*pGL-CDX2-1313-R*	ATCTGCAGGGGATGGGGTACCGGCCAGTTAGGC	
*pGL-CDX2-1992-R*	ACTGGGGGAGACTGGAGGTACCGGCCAGTTAGGC	
*pGL-CDX2-F*	AGCATGGTGAGGTTTTGAGCTCGCTAGCCTCGAG	
*pGL-TEAD4-208-R*	CAAGGGCAGGTCATCGGTACCGGCCAGTTAGGC	
*pGL-TEAD4-333-R*	AAGTACCATGGATGTGGTACCGGCCAGTTAGGC	
*pGL-TEAD4-589-R*	AAGTGTCCATGGACAGGTACCGGCCAGTTAGGC	
*pGL-TEAD4-989-R*	AGGTCAAATCGGAGCTGGTACCGGCCAGTTAGGC	
*pGL-TEAD4-1542-R*	TGACCAACCCCTCCTGGTACCGGCCAGTTAGGC	
*pGL-TEAD4-1998-R*	GAGAACAAAACCCCGGGTACCGGCCAGTTAGGC	
*pGL-TEAD4-F*	GTGCTTTCTGCCCACTGAGCTCGCTAGCCTCGAG	

### Transient transfection and luciferase assay

pTr cells were seeded in 96-well plates and transfected overnight with 100 ng/well of *CDX2* or *TEAD4* promoter-luciferase plasmids using Lipofectamine 3000 (Thermo Fisher Scientific, Waltham, MA, USA). After 48 h, luciferase activity was measured using the Steady-Glo Assay System (Promega, Madison, WI, USA) and detected with a Synergy4 plate reader (Bio-Tek, Winooski, VT, USA), following the manufacturer’s protocol.

### Development of a stable pTr-*CDX2*/*luc* luciferase reporter cell line

Based on previous transfection results, the *CDX2* promoter fragment with the highest fluorescence intensity (1,313 bp) was cloned into the pGF-mCMV-EF1-Neo lentiviral luciferase vector (System Biosciences, Palo Alto, CA, USA) using In-Fusion Cloning (Vazyme, Nanjing, China). HEK 293 T cells were transfected with the *CDX2* recombinant vector, PasPx2, and pMD2.g to produce pseudolentiviral particles, which were collected at 48 h post-transfection. For transduction, pTr cells were exposed to virus in DMEM medium for 6 h, followed by G418 selection. Single-cell clone with the highest luciferase activity (pTr-*CDX2*/*luc*) was prepared for the introduction of Renilla luciferase and *TEAD4* promoter.

### Development of a stable pTr-*CDX2*/*luc*-*TEAD4*/*Rluc* double luciferase reporter cell line

A 989-bp *TEAD4* gene promoter was cloned into the pLV-puro lentiviral Renilla luciferase reporter vector. Pseudolentiviral particles were generated by transfecting HEK 293 T cells in a 10-cm dish with recombinant *TEAD4* promoter lentivector, PasPx2, and pMD2. Following concentration, the pseudolentiviral particles were used to infect pTr-*CDX2*/*luc* cells. Transduced pTr-*CDX2*/*luc* cells were cultured in complete DMEM medium containing 1.20 μg/mL puromycin for a week of selection, with regular medium changes every 2–3 d. After 2 weeks, individual cell clones were expanded and assessed for Renilla luciferase activity. The cell clones exhibiting the highest luciferase activity were designated as pTr-*CDX2*/*luc*-*TEAD4*/*Rluc*, intended for subsequent high-throughput screening.

### High throughput screening of amino acids and their metabolites for pTr-*CDX2*/*luc*-*TEAD4*/*Rluc*

pTr-*CDX2*/*luc*-*TEAD4*/*Rluc* cells were seeded at 1 × 10^4^ cells/well in 96-well white plates with DMEM medium overnight. We prepared a nutritional library of 85 amino acids and metabolites, dissolving them in either DMSO or water. The concentration range for each substance was set from 10 µmol/L to 10 mmol/L (Additional file 1). For each substance, three different concentrations (high, medium, and low) were tested, and the treatment duration was 24 h.

After the treatment, luciferin was added to measure luciferase activity. Following the reaction, a stop solution was added, and coelenterazine was then added to measure Renilla luciferase activity. The luminescence was measured using a Synergy4 plate reader and the Dual Luciferase Reporter Assay Kit (Vazyme, Nanjing, China).

To identify positive metabolites, a *Z*-score was calculated using Z = (*x* − *µ*)/*σ*, where *x* is the relative luciferase activity of a metabolite, *µ* is the mean, and *σ* is the standard deviation of all tested compounds [22, 23]. Amino acids or their metabolites with a Z-score ≥ 2.0 were considered hits.

### RNA extraction and real-time PCR analysis

RNA was extracted from pTr and pEECs cells using TRIzol reagent (Invitrogen, USA) per the manufacturer’s instructions, with quality and quantity assessed via a Nanodrop (Thermo Fisher Scientific, Waltham, MA, USA). Reverse transcription was carried out using the PrimerScript RT Reagent Kit (Takara, Kyoto, Japan). Specific primers for real-time PCR were designed using Primer Premier 6.0 (Table S1). cDNA was amplified for gene expression analysis via SYBR Green real-time PCR (Takara, Kyoto, Japan), with the 2^−∆∆Ct^ method used for quantification, normalizing to β-actin.

### RNA interference

Small interfering RNA (siRNA) was utilized to knock down *CDX2* and *TEAD4,* and the siRNA sequences are listed in Table S2. Cells were transfected with siRNA upon reaching 70%–80% confluence, following the manufacturer's protocols. Specifically, pTr cells were transfected with either the targeting siRNA or control siRNA using Lipofectamine 3000 Reagent for 48 h.

### Response surface methodology in high-throughput screening cell model

Response surface methodology was employed to optimize candidate amino acid system—lysine, methionine, tryptophan, and arginine—using relative luciferase activity and relative Renilla luciferase activity as response variables within a central composite design. The determination of design levels was informed by preliminary laboratory experiments and a review of existing literature. Twenty-five amino acid combinations were replicated six times at the central point (Table S3).

After obtaining the relative luciferase and Renilla luciferase activity values for each combination using the high-throughput screening cell model, the data were analyzed and optimized using Design-Expert 13 software to determine the optimal concentration ratio of the candidate amino acid formulation.

### Cell adhesion assay

pTr cells were cultured in DMEM containing the candidate amino acid system (AAS) for 24 h, labeled with 5,6-carboxyfluorescein diacetate succinimidyl ester (CFSE) (Invitrogen, Carlsbad, CA, USA), and then seeded onto pEEC monolayers. After 1 h of incubation at 37 °C, the non-adherent (unattached) pTr cells were removed by gently washing the pEEC monolayers three times with PBS. The wash solutions, containing the unattached cells, were collected and quantified using a Scepter cell counter (Millipore, Burlington, MA, USA). The adhesion rate was calculated by subtracting the number of unattached cells from the total number of seeded pTr cells, and then expressed as the percentage of attached cells relative to the initial seeding density.

### Animals and experimental design

The animal procedures described in this study were approved by the Institutional Animal Care and Use Committee of China Agricultural University (AW92804202-1-2). Animal experiments were conducted at the Fengning Pig Research Center of China Agricultural University (Chengde Jiu Yun Academician Workstation), a subsidiary of Nongmu Share Co., Ltd.

#### Exp. 1

To explore the optimal amino acid profile in early gestation feed for sows, response surface methodology was employed to optimize combinations of candidate amino acids, including lysine, methionine, tryptophan, and arginine. Unlike in the cell experiments, arginine was replaced with *N*-carbamoyl glutamate (NCG), which is the activator of endogenous arginine synthesis and much more cost-effective compared to direct arginine supplementation [24]. These amino acids were evaluated using litter size as the response variable within a Central Composite Design [25]. The determination of design levels was based on preliminary laboratory experiments and a review of existing literature (Table S4). This study utilized 400 White × Landrace crossbred sows with similar parities, body weight, and backfat thickness. The sows were randomly assigned to 20 treatment groups, with 20 sows per group. From d 1 to d 28 of pregnancy, each group was fed the respective experimental diet, which included different levels of amino acids supplemented into the basal diet, as specified in Table S5. From d 28 of pregnancy until delivery, all sows were fed the same basal diet. After farrowing, the total number of piglets born, the number of liveborn piglets per litter, and their birth weights were recorded.

#### Exp. 2

To verify whether maternal supplementation with specific amino acid system (AAS) during early pregnancy could enhance the reproductive performance of sows, this study employed 40 White × Landrace crossbred sows with similar parities, body weight and backfat thickness. The control group received basal diet from weaning to d 28 of pregnancy. The treatment group, on the other hand, was provided with experimental diet supplemented with amino acids (0.15% methionine, 0.10% tryptophan, and 0.1% NCG) in addition to the basal diet, to fulfill the candidate amino acid system optimized through the response surface and cell model. From d 28 of pregnancy until delivery, all sows were fed the same basal diet. After farrowing, the number of born and liveborn piglets per litter, and birth weight were recorded.

Placental tissue collection: At the time of farrowing, the umbilical cord of the newborn piglets was tied with a short cotton thread and labeled with a number tag to ensure that each piglet was matched with the corresponding placenta. After the placenta was expelled, approximately 5 g of placental tissue (3 to 5 cm from the umbilical cord insertion point) was collected and rapidly frozen in liquid nitrogen.

### Transcriptome analysis

#### Embryo collection and culture

ICR mice were used as the source of embryos. Adult ICR mice were mated, and on the following day, zygotes were collected from healthy females. To ensure sample reliability, multiple females were used for each collection. We used KSOM medium containing the amino acid system (Lys:Met:Trp:Arg = 1:0.45:0.12:1.60, Lys = 0.25 mmol/L), and embryos were incubated at 37 °C in a 5% CO_2_ atmosphere for 72 h until they reached the blastocyst stage, The embryo culture method followed the procedures described in the literature [26]. For each experiment, the collected embryos were divided into two treatment groups with three replicates per group, each replicate consisting of 15–20 embryos. For transcriptome analysis, 5–8 blastocysts from each replicate were pooled as one sample. Embryo development was monitored by morphological evaluation, and the blastocyst formation rate was compared between the treatment groups.

#### RNA extraction and sequencing

Blastocysts were harvested, and total RNA was extracted using TRIzol reagent and subsequently treated with RNase-free DNase I (TaKaRa, Kyoto, Japan). RNA integrity and quality were assessed using 1% agarose gel electrophoresis, the Agilent 2100 Bioanalyzer (Agilent Technologies, Santa Clara, CA, USA), and a NanoDrop spectrophotometer (Thermo Fisher Scientific, Waltham, MA, USA). Sequencing libraries were then prepared and sequenced on an Illumina Novaseq 6000 platform (Beijing Allwegene Technology, Beijing, China). Gene expression levels were quantified using FPKM, and differential expression analysis was performed using DESeq in R. Genes with adjusted *P* < 0.05 were considered significantly differentially expressed. GO enrichment analysis was performed to assess the molecular functions and cellular components of the differentially expressed genes (DEGs), with ReviGO used to reduce redundancy. KEGG pathway enrichment analysis was also conducted, with significance set at *P* < 0.05.

### Statistical analysis

All experiments were repeated at least three times, and the values were presented as the means ± SEM. One-way ANOVA was utilized for analysis involving more than two groups, followed by Tukey's multiple comparisons, with a significance threshold set at *P* < 0.05.

## Results

### Selection of appropriate *CDX2 and TEAD4* gene promoter region constructs to establish a stable cell line

The pTr cell line, derived from 12-day porcine blastocysts, was chosen due to its robust growth, stable phenotype, and close association with key events in early embryonic development such as implantation and placental formation[27]. *CDX2* and *TEAD4* play crucial roles in the development of the embryonic trophoblast [28, 29]. *CDX2* is crucial for the specification of the trophectoderm, while *TEAD4* is important for maintaining trophoblast stem cell characteristics and regulating downstream genes involved in trophoblast proliferation and differentiation. Our results show that knocking down *CDX2* and *TEAD4* significantly reduces the expression of *GATA3* and *uPA*, both of which are crucial markers of trophoblast function (Fig. S1). To create a sensitive and quantitative screening system, we employed the promoter regions of *CDX2* and *TEAD4* to drive luciferase reporter expression.

**Fig. 1:**
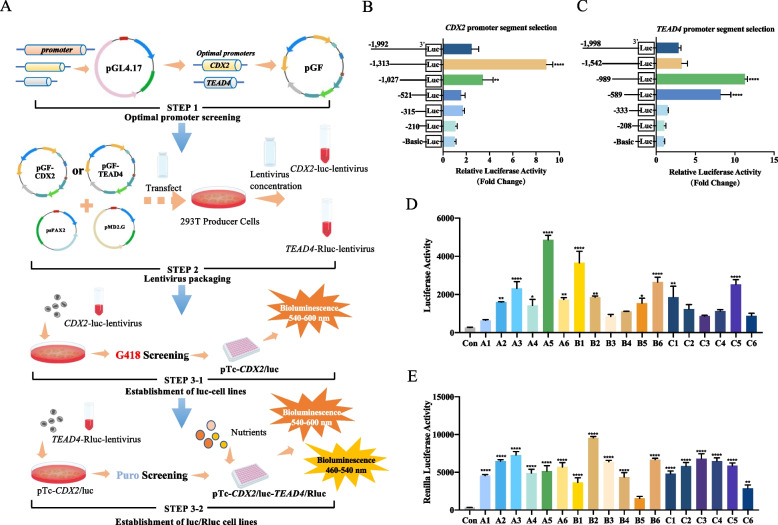
Optimization of stable pTr-*CDX2*/luc-*TEAD4*/Rluc dual luciferase report cells for high-throughput screening. **A** Construction of a high-throughput screening cell model. **B** and **C** *CDX2*/*TEAD4* gene promoter constructs of different lengths were cloned into pGL4.17 Luciferase reporter vector and transfected into pTr cells, followed by incubation for 48 h. The fold change in Luciferase activity of the cells transfected with each *CDX2*/*TEAD4* promoter construct relative to that of the cells transfected with the promoter-less basic vector is shown. **D** and **E** Luciferase/Renilla activity in each cell clone (A1–C6). Data were expressed as means ± SEM. ^*^P < 0.05, ^**^P < 0.01, ^***^P < 0.001, ^****^P < 0.0001

Figure 1A depicted a flow diagram illustrating the construction of a high-throughput screening cell model. To identify the optimal promoter fragments providing the highest luciferase activity, seven different lengths of the *CDX2* promoter regions were cloned into the pGL4.17 luciferase reporter vector. Luciferase assays revealed that the activity of the *CDX2* promoter depended on its length. Compared to the absence of the promoter, the 1,313 bp promoter segment led to an 8.89-fold increase in luciferase activity (Fig. 1B). Therefore, the 1,313-bp *CDX2* gene promoter segment was selected to drive luciferase reporter gene expression and utilized for subsequent stable cell line development. Further investigation indicated that two promoter regions (521–1,027 bp and 1,027–1,313 bp) upstream of the *CDX2* start codon exhibited significantly increased luciferase activity, suggesting the presence of consensus binding sites for critical transcription factors in these regions. Conversely, the inclusion of the segments (1,313–1,992 bp) resulted in clear suppression of luciferase activity, indicating the existence of binding sites for a negative regulator.

Similarly, seven different lengths of the *TEAD4* promoter regions were cloned into the pGL4.17 luciferase reporter vector. Luciferase assays demonstrated that compared to the absence of the promoter, the 989-bp *TEAD4* promoter segment led to an 11.29-fold increase in luciferase activity (Fig. 1C). Furthermore, two promoter regions upstream of the *TEAD4* start codon (333–589 bp and 589–989 bp) exhibited significantly increased luciferase activity, suggesting the presence of consensus binding sites for critical transcription factors in these regions. Conversely, the inclusion of the segment (989–1,542 bp) resulted in clear suppression of luciferase activity, indicating the presence of binding sites for a negative regulatory factor.

### Establishment of a cell-based high-throughput screening assay to identify *CDX2/TEAD4*-inducing amino acid metabolites

To establish a luciferase reporter cell line regulated by the *CDX2* gene promoter, we inserted the 1,313-bp *CDX2* promoter construct showing the highest fold increase into the lentiviral luciferase reporter vector pGF-mCMV-Neo. Pseudoviruses were generated in 293 T cells and utilized to infect pTr cells. Following a 2-week selection period with G418 medium, surviving cells underwent limiting dilution in 24-well plates. Individual cell clones were then expanded gradually, and their luciferase activity was evaluated. Among the 18 analyzed cell clones, clone A5 exhibited the highest luciferase activity (Fig. 1D). Consequently, we designated this stable cell clone as pTr-*CDX2*/*luc* and selected it for further experimentation.

To improve the precision and stability of high-throughput screening, we introduced the *TEAD4* gene promoter, the upstream gene of *CDX2,* along with the Renilla luciferase reporter gene. Similarly, to establish a stable luciferase reporter cell line driven by the *TEAD4* gene promoter, we cloned the 989-bp *TEAD4* promoter construct exhibiting the highest fold increase into the lentiviral Renilla luciferase reporter vector pLV. Pseudoviruses were then produced in 293 T cells and used to infect pTr-*CDX2*/*luc* cells. Following a 2-week selection period with Puromycin medium, surviving cells underwent limiting dilution in 24-well plates. Individual cell clones were gradually expanded, and their Renilla luciferase activity was assessed. Among the 18 cell clones analyzed, clone B2 exhibited the highest Renilla luciferase activity (Fig. 1E). Therefore, we designated this stable cell clone as pTr-*CDX2/luc*-*TEAD4/Rluc* for subsequent high-throughput screening experiments.

### High throughput screen for amino acids and their metabolites that regulated early embryonic development

**Fig. 2:**
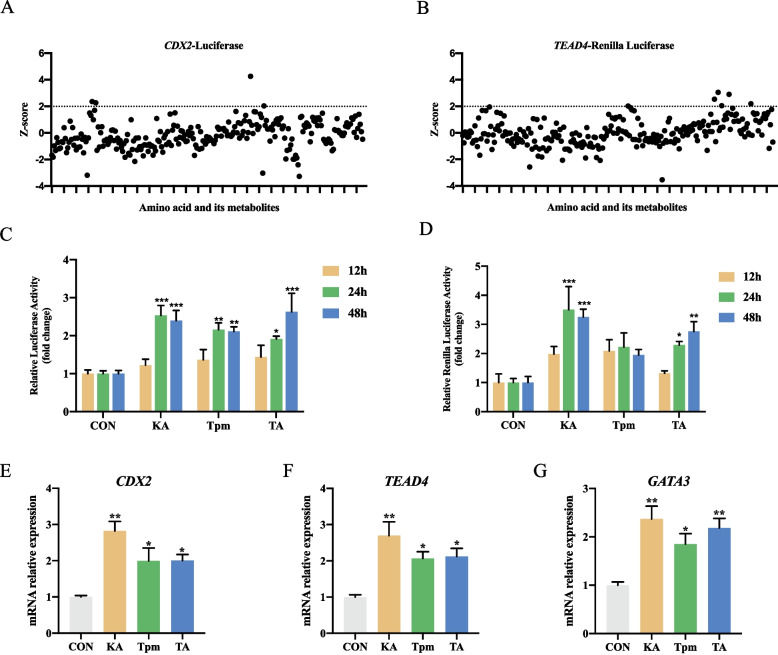
High throughput screening of amino acids and their metabolites. **A** and **B** Z-scores of 85 amino acid metabolites following a primary screening. **C** and **D** Time-dependent response of pTr-*CDX2*/luc-*TEAD4*/Rluc to three screened nutrients. **E**–**H** Impact of selected amino acid metabolites on early embryonic development-related genes. Data were presented as means ± SEM. ^*^P < 0.05, ^**^P < 0.01, ^***^P < 0.001, ^****^P < 0.0001

To identify amino acids and their metabolites capable of inducing the expression of *CDX2* and *TEAD4*, we conducted a high throughput screening of 85 different amino acid and their metabolites (each at three concentrations) in a 96-well plate format. Figure 2A and B illustrated the Z-scores for all metabolites. By applying a Z-score threshold of 2.0, we identified a total of 6 hits, including 4 compounds capable of inducing *CDX2* expression and 5 compounds capable of inducing *TEAD4* expression (Table 2). Notably, among the 6 compounds, 3 of them concurrently activated the expression of both *TEAD4* and *CDX2*. These compounds were kynurenic acid (KA), tryptamine (Tpm), and taurine (TA).

**Table 2:** The Z-scores and major functions of 6 hits from primary screening of the nutrient library

Compound	Z score (luc)	Z score (Rluc)	Major function
Kynurenic acid	4.25	3.05	Modulates immune responses and oxidative stress protection crucial for early embryonic development
Taurine	2.36	2.04	Supports embryonic development and protects cells from oxidative stress during early development.
Tryptamine	2.27	2.90	Influences cell signaling pathways that are crucial for embryonic development.
L-Arginine	1.70	2.19	Enhances placental blood flow and supports fetal growth during pregnancy.
L-Methionine	2.03	1.63	Essential for DNA methylation and gene expression regulation during embryogenesis.
S-Adenosyl-L-homocysteine	1.51	2.01	Regulates methylation reactions that are critical for DNA synthesis and early embryonic development.

To further validate the ability of the identified metabolites to induce *CDX2* and *TEAD4* expression, we conducted a time dependent experiment on stable pTr cells. The results indicated that treatment with kynurenic acid, tryptamine, and taurine for 24 h and 48 h significantly enhanced the luciferase activity of *CDX2*, with no effect observed at 12 h of treatment (Fig. 2C). Regarding *TEAD4*, treatment with kynurenic acid and taurine for 24 h and 48 h significantly promoted luciferase activity, with no effect observed at 12 h of treatment (Fig. 2D). Furthermore, we analyzed the effects of these amino acid metabolites on the expression of early embryonic development-related genes in pTr cells using RT-qPCR (Fig. 2E–G). As expected, all three compounds induced the expression of *CDX2* and *TEAD4*, as well as the downstream gene *GATA3*.

### Optimization of amino acid system by response surface methodology in high-throughput screening cell model

**Fig. 3:**
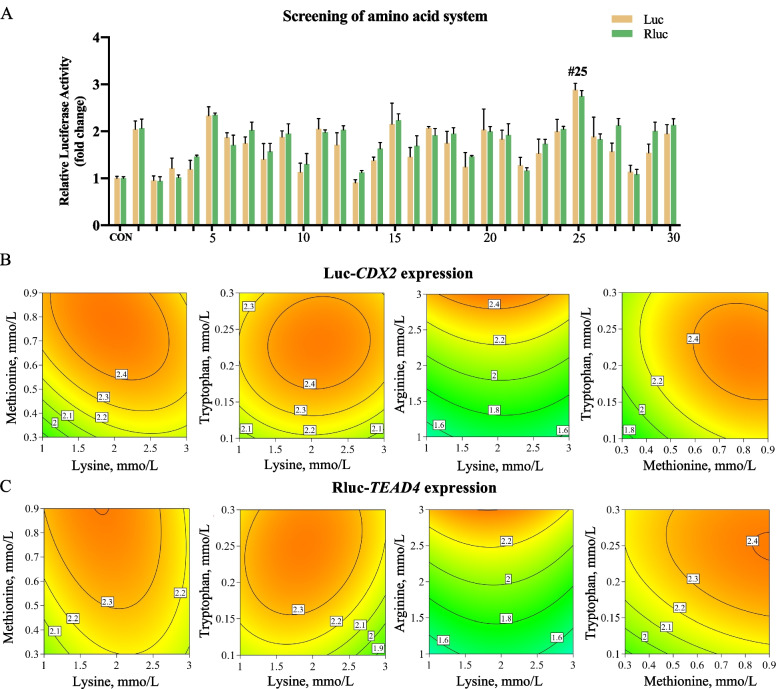
Response surface methodology of *CDX2* and *TEAD4* expression with optimal amino acid system. **A** High-throughput screening of different amino acid combinations for the activation of *CDX2* and *TEAD4*. **B** and **C** Relationship between various amino acid concentrations and relative luciferase activity and relative Renilla luciferase activity. #25 represents the 25th amino acid combination medium

The six identified hits were closely related to methionine, arginine, and tryptophan (Table 2), suggesting a potential link between these amino acids and early embryonic development. Using the response surface methodology in combination with high-throughput screening cell models, we designed the composition of each amino acid to explore the effects of their ratios and concentrations on early embryonic development. Following the combination of selected amino acids, we determined relative luciferase (luc) and Renilla luciferase (Rluc) activities (Fig. 3A). For luciferase, the 25th formulation (lysine = 2.0 mmol/L, methionine = 1.0 mmol/L, tryptophan = 0.5 mmol/L, and arginine = 4 mmol/L; luc = 2.883) demonstrated the most optimal promoting effect. Similarly, the 25th formulation exhibited the most optimal promoting effect for Renilla luciferase activities.

Figure 3B and C presented a response surface plot illustrating the complex relationship between independent variables (various amino acid contents) and relative luciferase activity and relative Renilla luciferase activity. Anova results indicated that arginine significantly influenced the response variables of luc and Rluc (*P* < 0.001). The experimental results were fitted to a well-fitted quadratic model, and the lack of fitness test (*P* > 0.05) was not significant. Furthermore, Fig. 3B and C illustrated that arginine positively influenced luc, with its increased value leading to an increase in luc, indicating elevated expression of the *CDX2*. Lysine, methionine, and tryptophan exhibited a biphasic effect on luc, where luc initially increased with their values and then decreased. This suggested that within an appropriate range, these three amino acids also had a promoting effect on *CDX2*. As expected, for Rluc, a similar trend to luc was observed, as *TEAD4* is an upstream regulatory gene of *CDX2*.

**Fig. 4:**
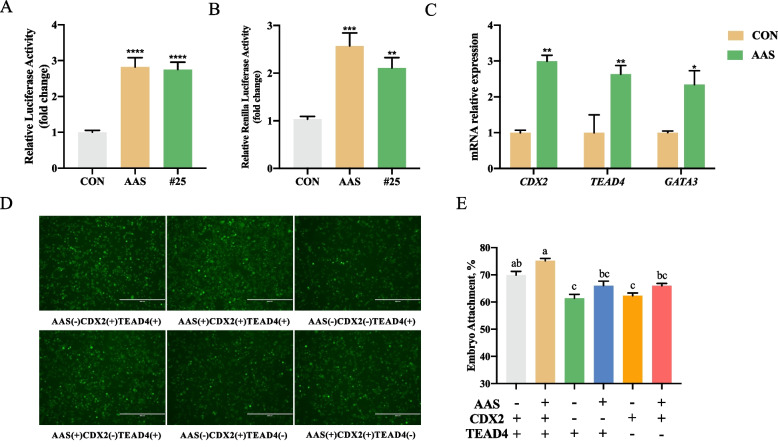
Validation of candidate amino acid system through in vitro experiments. **A** Effect of candidate amino acid system and #25 amino acid combination on *CDX2* promoter expression. **B** Impact of candidate amino acid system and #25 amino acid combination on *TEAD4* promoter expression. **C** Influence of candidate amino acid system and #25 amino acid combination on gene expression of *CDX2*, *TEAD4*, and *GATA3*. **D** and **E** Effects of candidate amino acid system and *CDX2*, *TEAD4* interference on in vitro embryo adhesion rate. Data were presented as means ± SEM.^*^P < 0.05, ^**^P < 0.01, ^***^P  < 0.001, ^****^P < 0.0001. ^a^^–^^c^Different letters represented differences between groups. #25 represents the 25th amino acid combination medium

To optimize the amino acid combination, we employed regression analysis and response surface modeling. Figure 4 shows the derived candidate amino acid system for the expression of *CDX2* and *TEAD4*. The optimal formulation was identified as lysine = 1.87 mmol/L, methionine = 0.82 mmol/L, tryptophan = 0.23 mmol/L, and arginine = 3 mmol/L, with the ratio of 1:0.43:0.12:1.60, the response values for luc and Rluc reached their peaks at 2.856 and 2.797, respectively (Table S6).

### Candidate amino acid system promoted the expression of genes related to early embryonic development and embryo adhesion

To validate the accuracy of the candidate amino acid system optimized through response surface methodology, we assessed their effects on key genes involved in early embryonic development. Compared to the control group, the candidate amino acid system significantly increased the relative luciferase activity and relative Renilla luciferase activity, outperforming the effects of the 25th formulations (Fig. 4A and B). Additionally, at the mRNA level, the candidate amino acid system significantly elevated the expression levels of *CDX2*, *TEAD4*, and *GATA3* (Fig. 4C).

To verify whether the candidate amino acid system promoted embryo adhesion, we established an in vitro adhesion model using pTr and pEECs cells. As shown in Fig. 4D and E, the embryo adhesion rate was significantly increased after treatment with the candidate amino acid system in pTr cells. To investigate whether the enhancement of embryo adhesion by the candidate amino acid system was related to the CDX2/TEAD4 pathway, we individually interfered with *CDX2* and *TEAD4*. The results showed that after interfering with *CDX2* and *TEAD4*, the embryo adhesion rate was significantly decreased compared to the control group. Interestingly, after adding the candidate amino acid system, regardless of interfering with *CDX2* or *TEAD4*, the embryo adhesion rate was still significantly decreased. These data suggested that the *CDX2*/*TEAD4* pathway was an important pathway through which the candidate amino acid system enhanced embryo adhesion.

### Transcriptome analysis of in vitro cultured mouse blastocysts with candidate amino acid system

**Fig. 5:**
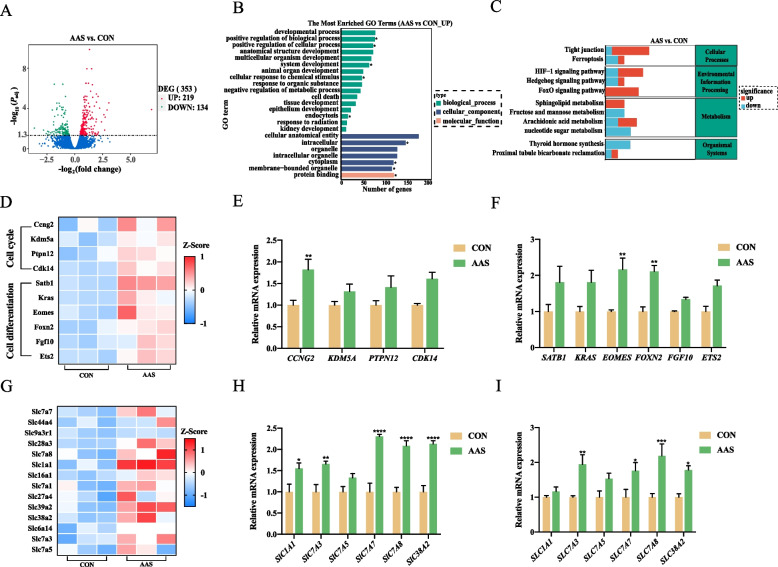
Effect of candidate amino acid supplementation on transcriptome profiling of mice blastocysts. **A** Volcano plot displaying differentially expressed genes in the candidate amino acid system and control groups. **B** Gene Ontology (GO) enrichment analysis of differentially expressed genes (DEGs) in the candidate amino acid system and control groups. **C** Kyoto Encyclopedia of Genes and Genomes (KEGG) enrichment analysis of DEGs in the candidate amino acid system and control groups. **D** Heatmap illustrating DEGs related to cell cycle regulation and differentiation. **E** Impact of candidate amino acid system supplementation on cell cycle regulation. **F** Impact of candidate amino acid system supplementation on cell cycle differentiation regulation. **G** Heatmap illustrating DEGs related to amino acid transporters in mouse blastocysts of the candidate amino acid system and control groups. **H** Impact of candidate amino acid system supplementation on amino acid transporter expression in pTr cells. **I** Influence of candidate amino acids supplementation on amino acid transporter expression in pig placenta. Data were presented as means ± SEM. ^*^P < 0.05, ^**^P < 0.01, ^***^P < 0.001, ^****^P < 0.0001

To further investigate the mechanisms by which candidate amino acid system influenced embryo development, we collected mouse zygotes and cultured them in media supplemented with candidate amino acids. After culturing the zygotes to the blastocyst stage, we observed that the blastocyst rate in the AAS group was numerically higher than that in the control group (56.6% vs. 59.4%), but no significant difference was found (Fig. S4). We then performed transcriptome sequencing analysis. Our analysis identified 219 upregulated and 134 downregulated differentially expressed genes (DEGs) in the blastocysts, as illustrated in the volcano plot (Fig. 5A). Gene Ontology (GO) analysis revealed that genes associated with developmental processes, system development, and protein binding were upregulated in the candidate amino acid group (Fig. 5B). Additionally, Kyoto Encyclopedia of Genes and Genomes (KEGG) pathway analysis highlighted significant enrichment in the Forkhead Box O (FoxO) signaling and tight junction pathways (Fig. 5C). The FoxO signaling pathway was closely linked to embryo development, while changes in tight junctions might be involved in subsequent embryo adhesion processes [30]. FoxO signaling exerts a profound influence on early embryo development by precisely regulating cell cycle progression and differentiation [31, 32]. In our heatmap, we illustrated the expression changes of differentially expressed genes involved in cell cycle regulation and differentiation (Fig. 5D). Notably, cell cycle negative regulators such as *Ccng2* and *Cdk14* were significantly upregulated in the candidate amino acid group, along with differentiation-related genes like *Eomes* and *Fgf10*. This suggested that candidate amino acid supplementation might promote early embryo development by activating the FoxO signaling pathway to regulate cell cycle progression and differentiation. These transcriptomic results were further validated in pTr cells using RT-PCR, confirming significant upregulation of CCNG2, EOMES, and FOXN2 in the candidate amino acid group (Fig. 5E and F). Furthermore, the solute carrier (SLC) superfamily, known for its role in substance transport, energy transfer, nutrient metabolism, and signal transduction, showed increased expression in the candidate amino acid group [33]. We displayed the mRNA levels of 14 SLC family DEGs in a heatmap, indicating that candidate amino acid supplementation might enhance nutrient transport and uptake in blastocysts (Fig. 5G). Specifically, we examined the expression of amino acid transporters within the SLC family and validated these findings through RT-PCR in pTr cells and porcine placental tissues. The results demonstrated significant upregulation of *SLC1A1*, *SLC7A3*, *SLC7A7*, *SLC7A8*, and* SLC38A2* in pTr cells (Fig. 5H). In placental tissues, *SLC7A3*, *SLC7A7*, *SLC7A8*, and *SLC38A2* were also significantly upregulated (Fig. 5I).

### Optimization of amino acid system by response surface methodology in sows

In addition to response surface methodology at the cellular level, we conducted response surface methodology of amino acid system (lysine, methionine, tryptophan, and arginine) in early gestation using 400 sows to validate the results obtained from high-throughput screening cell models and cellular-level response surface methodology. Unlike in the cell experiments, we replaced arginine with NCG. The response surface model's *P*-value was < 0.0001, indicating a good regression effect and strong significance. The lack-of-fit *P*-value was 0.7031, suggesting that the lack of fit is not significant and the multivariate quadratic regression model had a small error, making the experimental results valid and reliable. The regression coefficient* R*^2^ of the regression equation was 0.9713, the adjusted coefficient* R*_adj_^2^ was 0.9444, and the predicted coefficient *R*_pre_^2^ was 0.8939, with a difference less than 0.2, indicating that the regression equation had high credibility and reflected the true values well. The quadratic regression equation of the response surface fit well. From these results, we concluded that the model can be used to optimize and predict the ideal amino acid pattern in early gestation diets for sows.

**Fig. 6:**
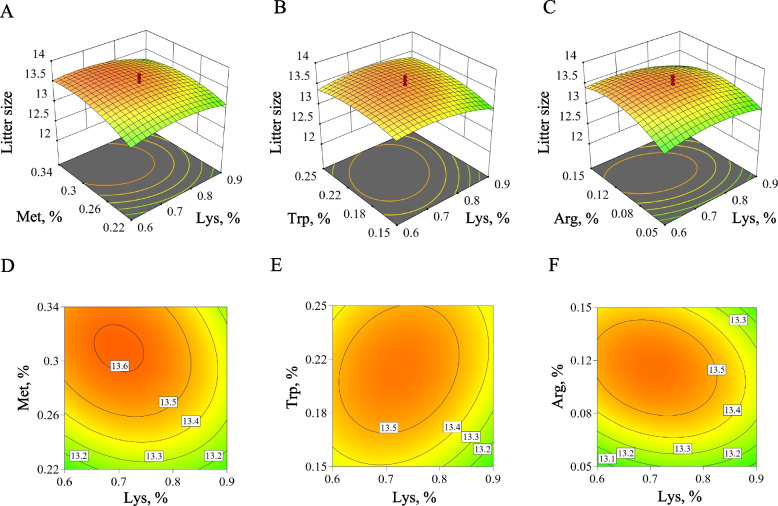
Response surface methodology of dietary amino acid system in early pregnancy of sows. **A** and **D** Relationship between dietary methionine and lysine concentrations and litter size. **B** and **E** Relationship between dietary tryptophan and lysine concentrations and litter size. **C** and **F** Relationship between dietary NCG and lysine concentrations and litter size

Figure 6 presented response surface plots showing the complex relationships between the independent variables (various amino acid levels in the diet) and the dependent variable (litter size). As shown in Fig. 6A and D, when lysine was at a lower level, litter size was gradually increased with the increase of methionine. At higher lysine levels, litter size was increased with methionine but then decreased, peaking at the methionine to lysine ratio of 0.44. Figure 6B and E showed that when lysine was between 0.68%–0.78% and tryptophan was between 0.19%−0.24%, the predicted litter size was higher, reaching the maximum at the tryptophan to lysine ratio of 0.29. As illustrated in Fig. 6C and F, litter size was initially increased and then decreased with the rise of NCG, and it ceased to increase when dietary NCG level exceeded 0.12%. We determined the optimal dietary amino acid system during early gestation to be 0.71% lysine, 0.32% methionine, 0.22% tryptophan, and 0.10% NCG, resulting in a litter size of 13.63 (Table S7). The optimized dietary amino acid system ratio, derived from the peripheral release of essential amino acids, was found to be 1:0.45:0.13 for lysine:methionine:tryptophan, which was similar with the results obtained from the cell model optimization.

### Effects of dietary supplementation with optimal amino acid system on the reproductive performance of sows

To evaluate the effectiveness of the optimal amino acid system and its impact on the reproductive performance of sows, the amino acid system was added to the sows' diet on day 28 of gestation. As shown in Table 3, dietary optimal amino acid supplementation during early gestation significantly increased litter size (12.20 vs. 13.90, *P* = 0.0144), live litter size (11.25 vs. 12.81, *P* = 0.0290), and litter weight (15.25 vs. 17.30, *P* = 0.0363). Additionally, optimal amino acid treatment had no significant effects on mean birth weight, sex distribution, offspring survival rate, stillbirth rate, malformation rate, or mummification rate (*P* > 0.05).

**Table 3:** Effect of optimal amino acid system diet in early pregnancy on reproductive performance of sows

Items	Control	Treatment	*P*-value
Number of sows, n	20	20	
Parity	2.20 ± 1.20	2.40 ± 1.31	0.6288
﻿Total piglets born per litter, n/litter	12.20 ± 2.52	13.90 ± 1.54	0.0144
﻿Total piglets born live per litter, n/litter	11.25 ± 2.68	12.81 ± 1.46	0.0290
﻿Litter birth weight of all piglets born alive, kg	15.25 ± 3.73	17.30 ± 1.92	0.0363

Values are presented as mean ± SEM

## Discussion

High early embryo mortality rates are key factors constraining sow reproductive performance [34, 35]. Improving embryo nutrition status and regulating early embryo development and implantation to enhance early embryo survival rates have attracted significant interest in the field [36, 37]. Current research indicates that maternal supplementation with amino acids such as arginine and methionine have a positive effect on early embryo development [38–40]. However, many potential amino acids and their metabolites that promote early embryo development remain unidentified, necessitating extensive cell or animal trials for screening and identification. To address this limitation, building on our previous laboratory research, we developed a dual-fluorescence high-throughput screening method by fusing the luciferase gene with the *CDX2* and *TEAD4* gene promoters separately and transducing lentivirus into pTr cells [41]. Utilizing this assay, we identified three amino acids and their metabolites with the potential to increase the expression of *CDX2* and *TEAD4*, demonstrating significant potential to enhance early embryo survival rates. These findings offered potential strategies for identifying other effective nutrients that can enhance sow reproductive performance.

Effective trophectoderm development is essential for early embryo growth and successful implantation [42]. The pTr cell line, derived from day 12 porcine embryos, serves as an invaluable model for studying early embryonic development due to its close association with trophectoderm differentiation [43, 44]. Both *CDX2* and *TEAD4* are highly expressed in the trophectoderm and are critical for its proper development [28, 45]; knockout of either gene results in embryonic developmental failure. *TEAD4* functions as an upstream regulator of *CDX2*, and embryos lacking *TEAD4* fail to express key trophectoderm markers, including *CDX2*, *ITGA7*, and *CDH3*, highlighting essential role of *TEAD4* in trophectoderm development [46]. Building upon preliminary laboratory research, we developed a dual-fluorescence high-throughput screening method that simultaneously evaluates the expression levels of *CDX2* and *TEAD4* by detecting fluorescence intensity [47, 48]. From the six amino acids or metabolites identified through this screening, kynurenic acid, taurine, and tryptamine stood out as notable regulators, promoting the expression of both *CDX2* and *TEAD4*. Interestingly, these metabolites are derived from or are analogs of methionine, tryptophan, and arginine, suggesting a close link between these amino acids and early embryonic development. Previous studies have indicated that optimizing *CDX2* and *TEAD4* expression can enhance blastocyst development. In this study, we confirmed that kynurenic acid, taurine, and tryptamine significantly promote the upregulation of *CDX2* and *TEAD4*. Moreover, downstream genes in the TEAD4 pathway, such as *GATA3*, are crucial for embryonic trophectoderm development, and we observed that kynurenic acid, taurine, and tryptamine also enhance *GATA3* expression.

Amino acids are pivotal in early embryo development, supporting essential processes such as cell division, proliferation, signal transduction, nutrient provision, and antioxidant activity [49, 50]. Intriguingly, the three substances identified in this study—kynurenic acid, taurine, and tryptamine—that concurrently enhance the expression of *CDX2* and *TEAD4* are all metabolites or analogs of methionine and tryptophan. Additionally, L-arginine was found to enhance the activity of the *TEAD4* promoter. In preliminary laboratory research, we examined the effects of arginine, including its precursor NCG, and methionine on early embryo development [51, 52]. However, this study further revealed the positive impact of tryptophan metabolites as well. To identify an optimal amino acid system that could more effectively promote trophectoderm development, we designed a response surface methodology experiment. We selected arginine, methionine, and tryptophan, with lysine included as a control. By leveraging response surface design in combination with high-throughput screening cell models, we efficiently screened and optimized this candidate amino acid system. Notably, our results highlight arginine as the most influential factor affecting luciferase and Renilla luciferase activity, underscoring its crucial role in regulating gene expression related to early trophectoderm development, particularly concerning the *CDX2* and *TEAD4*. Moreover, the biphasic effects observed for lysine, methionine, and tryptophan on luciferase activity suggest that their impact is nuanced and operates optimally within specific ranges, further enhancing the expression of* CDX2*. Similarly, *TEAD4*, as an upstream regulatory gene of *CDX2*, exhibited a response pattern similar to luciferase activity, which was expected.

Upon obtaining the optimized amino acid system, we validated its effects at the cellular level. Proper development of trophectoderm cells is crucial for successful embryo implantation, which is a key factor in ensuring early embryo survival [53]. Our findings showed that key genes involved in early trophectoderm development, including *CDX2*, *TEAD4*, and *GATA3*, were significantly upregulated in response to candidate amino acid system. Moreover, the in vitro embryo adhesion model, utilizing pTr and pEECs cells, serves as an important tool for studying how nutrients regulate embryo implantation. In our study, candidate amino acid system significantly enhanced embryo adhesion rates. Interestingly, when either *CDX2* or *TEAD4* was knocked-down, the embryo adhesion rate still decreased significantly even after the addition of the candidate amino acid system. This underscores the role of candidate amino acid system in promoting early trophectoderm development through the CDX2/TEAD4 pathway, confirming its potential as a regulator of key pathways involved in embryo adhesion.

To further understand the potential mechanisms by which candidate amino acid system influences early embryo development and implantation, we performed a transcriptome analysis on candidate amino acid system-treated blastocysts. While there are species-specific differences in certain aspects of embryo development, many key developmental signaling pathways are highly conserved across mammals [54]. The GO analysis revealed an enrichment of genes associated with developmental processes, system development, and protein binding, suggesting that amino acid system may enhance the overall developmental competency of embryos by modulating key biological processes critical for successful embryogenesis. Notably, the KEGG pathway analysis indicated a significant enrichment in the FoxO signaling pathway, which is known to play a pivotal role in regulating cell cycle progression, apoptosis, and cellular differentiation [30–32]. The upregulation of cell cycle negative regulators such as *Ccng2* and *Cdk14* within this pathway implied that amino acid system might influence developmental outcomes by finely tuning cell cycle. Specifically, it may slow down cell proliferation to ensure proper cell differentiation and the formation of embryonic structures. Furthermore, the upregulation of differentiation-related genes such as *Eomes* and *Fgf10* supported this hypothesis, suggesting that amino acid system not only impacted cell proliferation but also actively promoted cellular differentiation within the developing embryo. The dual role of FoxO signaling in regulating both cell cycle and differentiation aligns with the observed enhancement of embryo development in the amino acid system group. Additionally, previous studies have reported that amino acid supplementation affects the expression of placental nutrient transporters [55, 56]. In this study, the upregulation of genes within the SLC superfamily suggested that amino acid system group enhanced nutrient transport and uptake during early embryo development. This finding was corroborated by RT-PCR validation in pTr cells and porcine placental tissues, indicating that the amino acid system promotes early embryo development by upregulating nutrient transporter expression, thereby improving nutrient absorption.

Exploring optimal amino acid system only at the cellular level is insufficient. Extending response surface methodology to in vivo conditions, we utilized 400 sows to optimize the amino acid system during early pregnancy, thereby validating the results from the high-throughput screening cell model and cellular-level response surface methodology. Unlike the cell experiments, we replaced arginine with NCG, a feed additive that promotes the endogenous synthesis of arginine and can provides greater economic benefits compared to direct arginine supplementation [57]. As expected, the optimized amino acid system ratio of the feed, derived from the peripheral release of essential amino acids, was 1:0.45:0.13 for lysine:methionine:tryptophan, which was largely consistent with the results obtained from the cell model optimization. Notably, based on the response surface results, methionine and arginine emerged as more critical during early pregnancy in sows, with their levels exerting a greater impact on variations in litter size. Additionally, we found that the candidate amino acid system optimized at the cellular level, when converted and added to the sows' diet, significantly improved reproductive performance indicators, including increases in litter size, live litter size, and litter weight. These findings underscore the potential of optimal amino acid system as a feasible strategy to enhance reproductive performance in pigs, confirming the effectiveness of our combined high-throughput screening and response surface methodology.

## Conclusion

In conclusion, we successfully established a dual-fluorescence high-throughput screening cell model to efficiently identify potential nutrients that promote trophectoderm development. By combining response surface methodology with high-throughput screening experiments, we identified an optimal dietary amino acid system that enhanced early embryo development and implantation with 0.71% lysine, 0.32% methionine, 0.22% tryptophan, and 0.10% NCG. This innovative approach overcomes the limitations of traditional amino acid nutrition studies in sows, providing a more effective model for improving reproductive outcomes.

## Supplementary Information

Additional file 1: List of amino acids and their metabolite libraries.

Additional file 2: Fig. S1 Interference of *CDX2* and *TEAD4* on pTr cells. A–D Effects of interfering *CDX2* and *TEAD4* on *CDX2*, *TEAD4*, *GATA3* and *uPA* in pTr cells. Fig. S2 Expression of *CDX2* and *TEAD4* in Meishan pig embryos at day 12 of gestation. Fig. S3 Effects of candidate compounds on cell viability. Fig. S4 Effects of the amino acid system on mouse blastocyst rate.

Additional file 3: Table S1 Primer sequences used in real-time PCR. Table S2 SiRNA sequences used for this study. Table S3 Factors and levels of response surface test in cells. Table S4 Factors and levels of response surface test in sows. Table S5 Composition and nutrient profile of the AAS diets for sows. Table S6 Amino acids combination to achieve optimal response values (cell model). Table S7 Amino acids combination to achieve optimal response values (sows).

## Data Availability

The datasets used during the current study are available from the corresponding author on reasonable request.

## Abbreviations

AAS: Amino acid system
CDX2: Caudal Type Homeobox 2
CFSE: 5,6-Carboxyfluorescein diacetate succinimidyl ester
DEGs: Differentially expressed genes
GO: Gene Ontology
FoxO: Forkhead Box O
KA: Kynurenic acid
KEGG: Kyoto Encyclopedia of Genes and Genomes
luc: Luciferase
NCG: *N*-Carbamoyl glutamate
pTr: Porcine trophectoderm cell line
pEECs: Porcine endometrial stromal cell line
Rluc: Renilla luciferase
SLC: Solute carrier
TA: Taurine
TEAD4: TEA Domain Transcription Factor 4
Tpm: Tryptamine
