# Supplementary material for: Optimal amino acid system for early embryo development in sows based on response surface methodology and high-throughput screening cell models
Source: J Anim Sci Biotechnol. 2025 Apr 25;16:61. doi: 10.1186/s40104-025-01194-w (PMC12023682; doi:10.1186/s40104-025-01194-w)
Supplement: Supplementary file 2 — Additional file 2: Fig. S1 Interference of CDX2 and TEAD4 on pTr cells. A–D Effects of interfering CDX2 and TEAD4 on CDX2, TEAD4, GATA3 and uPA in pTr cells. Fig. S2 Expression of CDX2 and TEAD4 in Meishan pig embryos at day 12 of gestation. Fig. S3 Effects of candidate compounds on cell viability. Fig. S4 Effects of the amino acid system on mouse blastocyst rate. [file 40104_2025_1194_MOESM2_ESM.pdf]

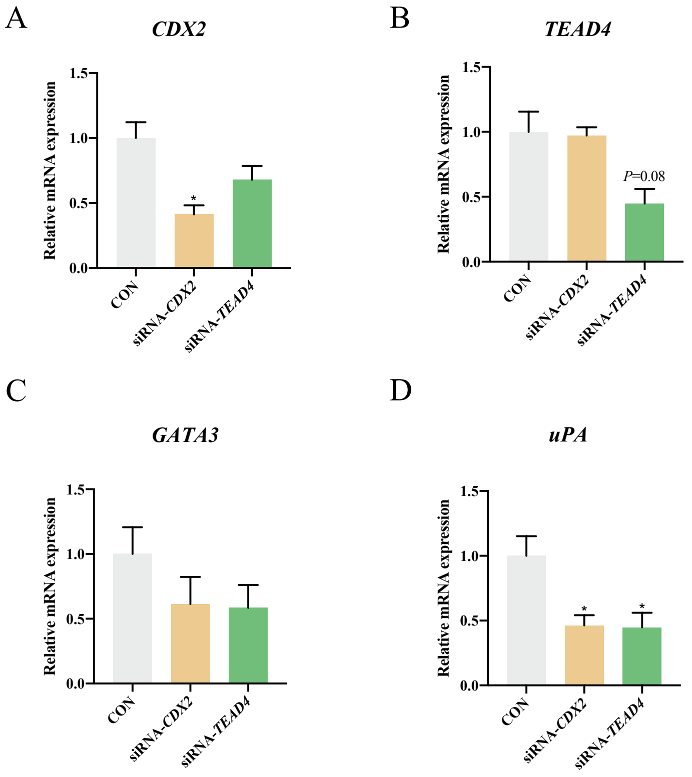

Fig. S1 Interference of *CDX2* and *TEAD4* on pTr cells. **A-D** Effects of interfering *CDX2* and *TEAD4* on *CDX2*, *TEAD4*, *GATA3* and *uPA* in pTr cells.

A

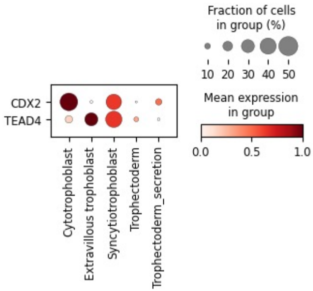

Fig. S2 Expression of *CDX2* and *TEAD4* in Meishan pig embryos at day 12 of gestation.

A

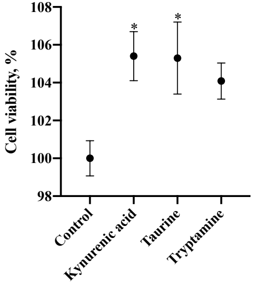

Fig. S3 Effects of candidate compounds on cell viability.

A

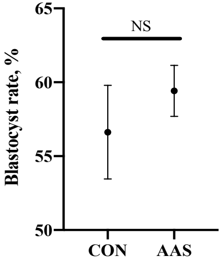

Fig. S4 Effects of the amino acid system on mouse blastocyst rate.
