# Supplementary material for: Optimal amino acid system for early embryo development in sows based on response surface methodology and high-throughput screening cell models
Source: J Anim Sci Biotechnol. 2025 Apr 25;16:61. doi: 10.1186/s40104-025-01194-w (PMC12023682; doi:10.1186/s40104-025-01194-w)
Supplement: Supplementary file 3 — Additional file 3: Table S1 Primer sequences used in real-time PCR. Table S2 SiRNA sequences used for this study. Table S3 Factors and levels of response surface test in cells. Table S4 Factors and levels of response surface test in sows. Table S5 Composition and nutrient profile of the AAS diets for sows. Table S6 Amino acids combination to achieve optimal response values (cell model). Table S7 Amino acids combination to achieve optimal response values (sows). [file 40104_2025_1194_MOESM3_ESM.docx]

**Table S1 ﻿**Primer sequences used in real-time PCR

| Gene name | Forward primer sequence (5'→3') | | Reverse primer sequence (5'→3') |
| --- | --- | --- | --- |
| *CDX2* | TGTGAGGGAGTGTTTCGGAC | AACGCCCCTTTTTCATCAGC | |
| *TEAD4* | GGCACCATTACCTCCAACGA | TAGGATGATCTTGCGCCGAC | |
| *GATA3* | CCCACCACCCTTCCAGTATG | TCCATGGGGTTTCAGCTTCG | |
| *PLAU* | GTCACCGGCCTGCTTATGAT | CATCTCCCCATGAGTGCTGG | |
| *CCNG2* | AGGGGTTCAGCTTTTCGGATT | AGTGTTATCATTCTCCGGGGTAG | |
| *KDM5A* | CACAGACCCGCTGAGTTTTAT | CTTCACAGGCAAATGGAGGTT | |
| *PTPN12* | ATGGAGCAAGTGGAGATCCTG | TCTCAATCGCATGAAGTCCCG | |
| *CDK14* | CTTTGTCCGAGAGTTTCAGCC | AGTTCCGGGTAGACATCTTTGT | |
| *SATB1* | CATGTTACCAGTTTTCTGCGTG | GTGAATAGCCTAGAGACAGCAA | |
| *KRAS* | CAAGAGCGCCTTGACGATACA | CCAAGAGACAGGTTTCTCCATC | |
| *EOMES* | GCGCATGTTTCCTTTCTTGAG | GGTCGGCCAGAACCACTTC | |
| *FOXN2* | GCCATCCTTTGGACCATCGT | TGGCGAGTGTTCAATAGCCATA | |
| *FGF10* | TTTGGTGTCTTCGTTCCCTGT | TAGCTCCGCACATGCCTTC | |
| *ETS2* | CCTGTCGCCAACAGTTTTCG | TGGAGTGTCTGATCTTCACTGA | |
| *SLC7A1* | TGGGCTTCATAATGGTGTCA | TGGTAGCGATGCAGTCAAAG | |
| *SLC7A3* | GCCATCCATTGTGATCTGCTT | GTGGTTCCCAATCAGGTTGTC | |
| *SLC7A5* | GGAAGGGTGATGTGTCCAATC | TAATGCCAGCACAATGTTCCC | |
| *SLC7A7* | TCGCTCTGGCACTCTACTCA | AGGCCACGTTGGTTAAGATG | |
| *SLC7A8* | AGGCTGGAACTTTCTGAATTAC | ACATAAGCGACATTGGCAAAGA | |
| *SLC38A2* | ATGAGTTGCCTTTGGTGATCC | ACAGGACACGGAACCTGAAAT | |
| *β-Actin* | CGGGACAACTGGGTGTACTG | AGTTGAAGGTGGTCTCGTG | |

**Table S2** SiRNA sequences used for this study

| **Items** | **Sense (5'→3')** | **Antisense (5'→3')** |
| --- | --- | --- |
| CDX2 | CGAAAGACAAAUACCGAGUTT | ACUCGGUAUUUGUCUUUCGTT |
| TEAD4 | GGCUUUGGACAAGCCCAUUTT | AAUGGGCUUGUCCAAAGCCTT |
| NC | UUCUCCGAACGUGUCACGUTT | ACGUGACACGUUCGGAGAATT |

**Table S3** Factors and levels of response surface test in cells

| **Level** | **Factor** | | | |
| --- | --- | --- | --- | --- |
|  | **A Lys, mmo/L** | **B Met, mmol/L** | **C Trp, mmol/L** | **D Arginine, mmol/L** |
| -2 | 0 | 0 | 0 | 0 |
| -1 | 1.5 | 0.5 | 0.25 | 1 |
| 0 | 3 | 0.1 | 0.5 | 2 |
| 1 | 4.5 | 1.5 | 0.75 | 3 |
| 2 | 6 | 2 | 0.1 | 4 |

**Table S4** Factors and levels of response surface test in sows

| **Level** | **Factor** | | | |
| --- | --- | --- | --- | --- |
|  | **A Lys, %** | **B Met, %** | **C Trp, %** | **D NCG, %** |
| -2 | 0.45 | 0.16 | 0.1 | 0 |
| -1 | 0.6 | 0.22 | 0.15 | 0.05 |
| 0 | 0.75 | 0.28 | 0.2 | 0.1 |
| 1 | 0.9 | 0.34 | 0.25 | 0.15 |
| 2 | 1.05 | 0.40 | 0.3 | 0.2 |

**Table S5** Composition and nutrient profile of the AAS diets for sows

| **﻿Ingredients** | **﻿Content, %** | **﻿Nutritional level** | **﻿Content, %** |
| --- | --- | --- | --- |
| Corn | 33.65 | ﻿Calculated values |  |
| Soybean meal | 2.00 | Dry matter | 88.95 |
| Broken rice | 9.10 | Crude protein | 11.89 |
| Sorghum | 10.00 | Crude fiber | 10.53 |
| Wheat flour | 3.00 | Acid detergent fiber | 12.1 |
| Wheat bran | 18.50 | Neutral detergent fiber | 24.76 |
| Soybean hulls | 15.50 | Gross Energy, kcal/kg | 3871 |
| Alfalfa meal | 6.00 | Net energy, kcal/kg | 2445 |
| Calcium hydrogen phosphate | 0.51 | Calcium | 0.64 |
| Stone powder | 0.25 | Total phosphorus | 0.51 |
| Choline chloride (50%) | 0.15 | Available phosphorus | 0.27 |
| Premix^1^ | 0.50 | Lysine | 0.71 |
| Salt | 0.30 | Methionine | 0.32 |
| L-Lysine hydrochloride (98.5%) | 0.13 | Threonine | 0.46 |
| L-Threonine | 0.06 | Tryptophan | 0.23 |
| NCG | 0.10 | Arginine | 0.60 |
| Methionine | 0.15 | Valine | 0.56 |
| Tryptophan | 0.10 | Isoleucine | 0.40 |
| Total | 100.00 |  |  |

^1^ Each kilogram of diet provides the following nutrients: zinc, 60 mg; iron, 95 mg; copper, 10 mg; iodine, 0.35 mg; selenium, 0.3 mg; manganese, 80 mg; vitamin A, 12,000 IU; vitamin D_3_, 2750 IU; vitamin E, 30 IU; vitamin K_3_, 2 mg; vitamin B_12_, 12 μg; vitamin B_2_, 6 mg; niacin, 40 mg; pantothenic acid, 12 mg; vitamin B_6_, 3 mg; biotin, 0.2 mg

**Table S6** Amino acids combination to achieve optimal response values (cell model)

| **Lys, mmol/L** | **Met, mmol/L** | **Trp, mmol/L** | **Arg, mmol/L** | **Relative Luciferase Activity** | **Relative Renilla Luciferase Activity** | **Desirability** |
| --- | --- | --- | --- | --- | --- | --- |
| 1.87 | 0.82 | 0.24 | 3.00 | 2.856 | 2.797 | 0.798 |

**Table S7** Amino acids combination to achieve optimal response values (sows)

| **Lys, %** | **Met, %** | **Trp, %** | **NCG, %** | **Litter size** | **Desirability** |
| --- | --- | --- | --- | --- | --- |
| 0.709 | 0.317 | 0.221 | 0.098 | 13.633 | 0.920 |
